# Investigation of health risk assessment and the effect of various irrigation water on the accumulation of toxic metals in the most widely consumed vegetables in Iran

**DOI:** 10.1038/s41598-022-25101-9

**Published:** 2022-12-02

**Authors:** Kiomars Sharafi, Borhan Mansouri, Abdullah Khalid Omer, Parnia Bashardoust, Gholamreza Ebrahimzadeh, Shokoufeh Sharifi, Tooraj Massahi, Hamed Soleimani

**Affiliations:** 1 Research Center for Environmental Determinants of Health (RCEDH), Research Institute for Health, Kermanshah University of Medical Sciences, Kermanshah, Iran; 2 Substance Abuse Prevention Research Center, Research Institute for Health, Kermanshah University of Medical Sciences, Kermanshah, Iran; 3 Department of Food Hygiene and Quality Control, Faculty of Veterinary Medicine, Urmia University, Urmia, Iran; 4 Department of Environmental Health Engineering, School of Public Health, Tehran University of Medical Sciences, Tehran, Iran; 5 Student’s Scientific Research Center, Tehran University of Medical Sciences, Tehran, Iran; 6 Department of Environmental Health Engineering, School of Public Health, Zabol University of Medical Sciences, Zabol, Iran; 7 Department of Agronomy, Faculty of Agriculture, Shahrekord University, Shahrekord, Iran; 8 Student Research Committee, Kermanshah University of Medical Sciences, Kermanshah, Iran

**Keywords:** Environmental sciences, Hydrology, Planetary science

## Abstract

The quality of irrigation water sources can significantly affect the concentrations of heavy metals (HMs) in cultivated vegetables. This study aimed to investigate the effect of various water resources, including treated wastewater effluent (TWE), river water (RW), and well water with chemical fertilizer (WW+F), on the accumulation of heavy metals (HMs) in the three most widely consumed edible vegetables (Coriander, Radish, and Basil) in Iran. A total of 90 samples of edible vegetables, 13 samples of irrigation water, and 10 soil samples were collected to determine HMs concentrations. Iron (Fe), Zinc (Zn), Copper (Cu), Manganese (Mn), Lead (Pb), Cadmium (Cd), Chromium (Cr), Nickel (Ni,) and Arsenic (As) were analyzed by inductively coupled plasma optical emission spectrometry (ICP-OES). Eventually, the Total Target Hazard Quotient (TTHQ) for the toxic metals of As, Pb, and Cd was determined. The results revealed that the TTHQ of toxic metals in vegetables was less than the allowable limits (TTHQ = 1). Also, TWE was the best irrigation water type since the HMs content of vegetables was low. By comparing the results with national and international standards, it can be concluded that the Gharasou RW for irrigation of edible vegetables was inappropriate.

## Introduction

Nowadays, the scarcity of suitable freshwater resources has become one of the most acute crises several countries face. Fast urbanization, population growth, and industrial developments have increased water demands in the last few decades, eventually leading to vast raw wastewater generation^1,2^. Various treatment methods are essential in obtaining large quantities of treated wastewater effluent (TWE). The mentioned limitations have made researchers use unconventional water resources (e.g., brackish water, municipal and industrial wastewater effluents) for various cultivation purposes^3^. Various agricultural cultivation methods include using groundwater combined with fertilizers, surface water such as river water, and treated wastewater effluents (TWE)^4,5^. All mentioned irrigation methods have their pros and cons.

Although the application of groundwater combined with chemical fertilizers for agricultural purposes might have lower microbial contamination, its limited resources and high HMs content in the used chemical fertilizers are among the most critical limiting factors^6,7^. Most surface water resources (especially rivers) are available in small quantities. Since most rivers accept raw and treated municipal and industrial wastewater, there is a high possibility of contamination via various pollutants, especially HMs^8,9^. TWE is considered the most suitable and available alternative source for irrigation and farming purposes. Although it is highly available and inexpensive, there is a high chance of contamination due to incomplete treatment processes, which can limit its application^10,11^.

Another critical environmental problem in recent years is the soil pollution caused by the heavy metals in chemical fertilizers or TWE used for agricultural purposes^12–14^. Various HMs (e.g., Cadmium (Cd), Arsenic (As), Chromium (Cr), Lead (Pb), and Zinc (Zn)) can enter the food chain via contaminated soils and water, reaching critical concentrations, causing harmful metabolites in the body, and having adverse effects on the living organisms^15,16^. Cadmium (Cd) has an atomic weight of 114.4, which can cause kidney damage, hypertension, mutagenicity, and carcinogenesis^17^. Diabetes, liver disease, cardiovascular disease, and various types of cancer are some ramifications caused by exposure to high contents of Arsenic (As) element^18^. Chromium (Cr) has an atomic weight of 24 with capacities of 2^+^ to 6^+^. International Agency for Research on Cancer (IARC) has classified Cr^+6^ into group 1 (carcinogenic to humans). Cr^+6^ at 10 mg/kg body weight can lead to liver necrosis, nephritis, and death^19^. Three sensitive organs of the hematopoietic, nervous, and renal systems of the human body can be threatened by Lead (Pb). Pb can have detrimental and severe adverse effects on the human body^20^. Although Zinc (Zn) is announced to be an essential element for human survival due to its effect on enzymes and protein production, exposure to high concentrations of Zn might lead to adverse health effects^21^.

As the main cultivational regions, the provinces of Hamedan, Kermanshah, Khuzestan, Lorestan, and Kurdistan, located in the west of Iran, supply the major portion of edible vegetables for the country^22,23^. Because of indiscriminate groundwater extraction by increasing the deep wells, our study area (Kermanshah province) is facing a groundwater resource shortage. Consequently, various irrigation sources are used in this region for farming purposes. The mentioned HMs in the irrigation water resources have become the environmentalist's primary concern; vegetables play a dominant role in people’s daily diet and contain high nutritional value^17^. Usually, farmers in the western regions of Iran do not pay attention to the amount and type of pollution that each irrigation source transmits to vegetables.

Since the chosen irrigation sources might contain HMs in high concentrations, and there is a possibility that the cultivated edible vegetables by these water sources could get contaminated by toxic HMs, and eventually cause acute and chronic health effects in consumers, a non-carcinogenic risk assessment was performed for HMs of Cd, As, Cr, Pb, and Zn.

Therefore, the present study was designed to investigate the following objectives:

- The effect of three different irrigation sources, including well water with chemical fertilizer (WW+F) (first source), TWE (second source), and river water (RW) (third source), on the concentration of heavy metals, including As, Cd, Pb, Cu, Fe, Zn, Cr, Mn, and Ni be investigated in three types of high-consumption vegetables in Iran (Coriander, Basil, and Radish).
- Determining each irrigation water source's effect on carcinogenicity and non-carcinogenicity risk of the studied heavy metals from the vegetables mentioned above for Iranian consumers.

## Materials and methods

### Irrigation of cultivated vegetables with different water sources

**Figure 1:**
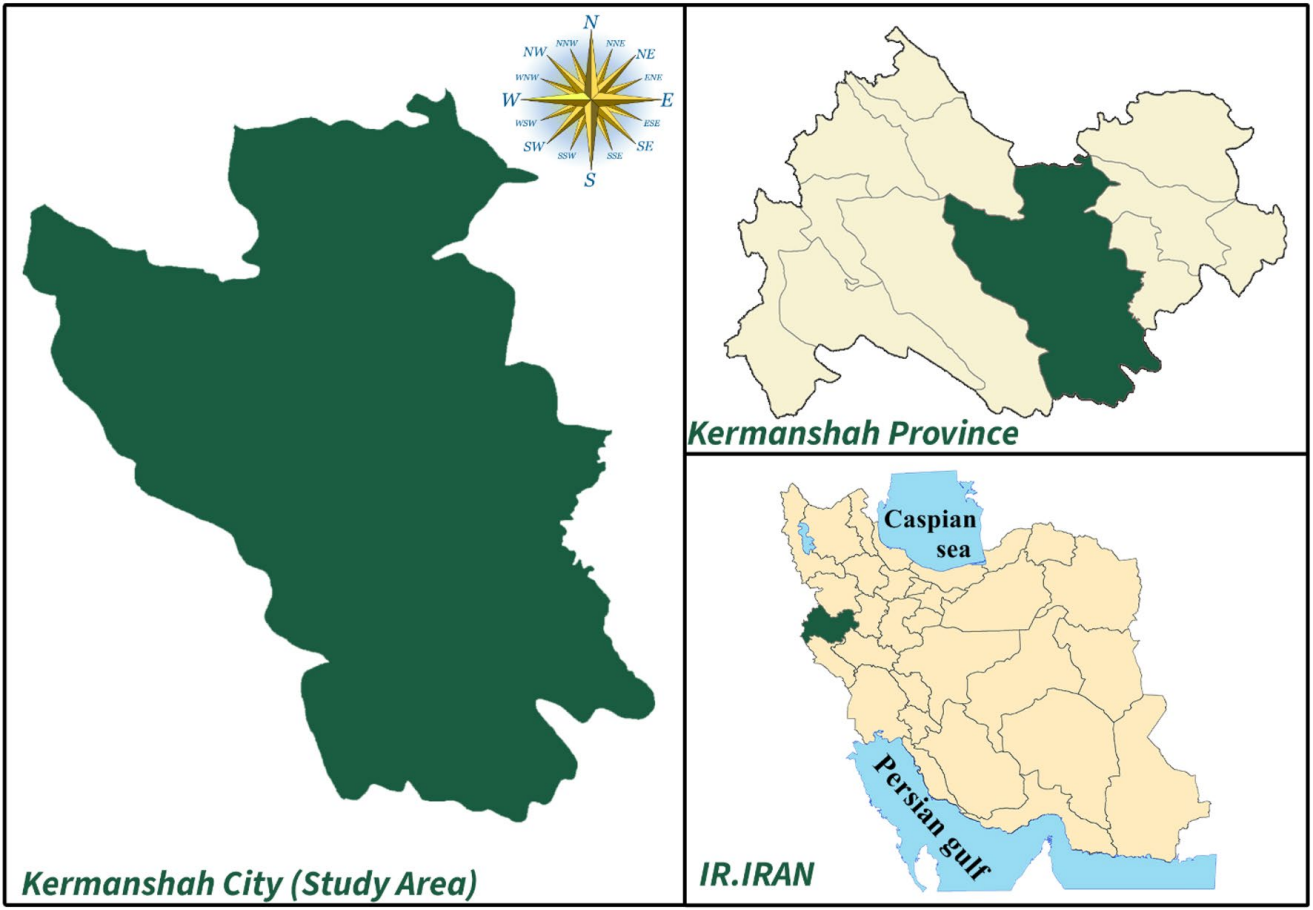
Location map of the study area.

The required cultivation site was prepared by coordinating and consultations with the Kermanshah Municipal Wastewater Treatment Plant. Figure 1 shows the location of Kermanshah city. Kermanshah is the ninth most populous city and one of the metropolises of Iran. The population of the Kermanshah metropolis amounted to 946,651 people in the 2015 census. The city of Kermanshah has a mild mountainous climate. The average annual temperature of Kermanshah city is about 14 °C, and the annual rainfall of this city is 456.8 mm. Geographically, this city is located at a latitude of 34.327715, a longitude of 47.077898, and a height of 1200 m above sea level. This study was carried out in a 36-square-meter plot of agricultural land adjacent to the Kermanshah wastewater treatment plant.

**Figure 2:**
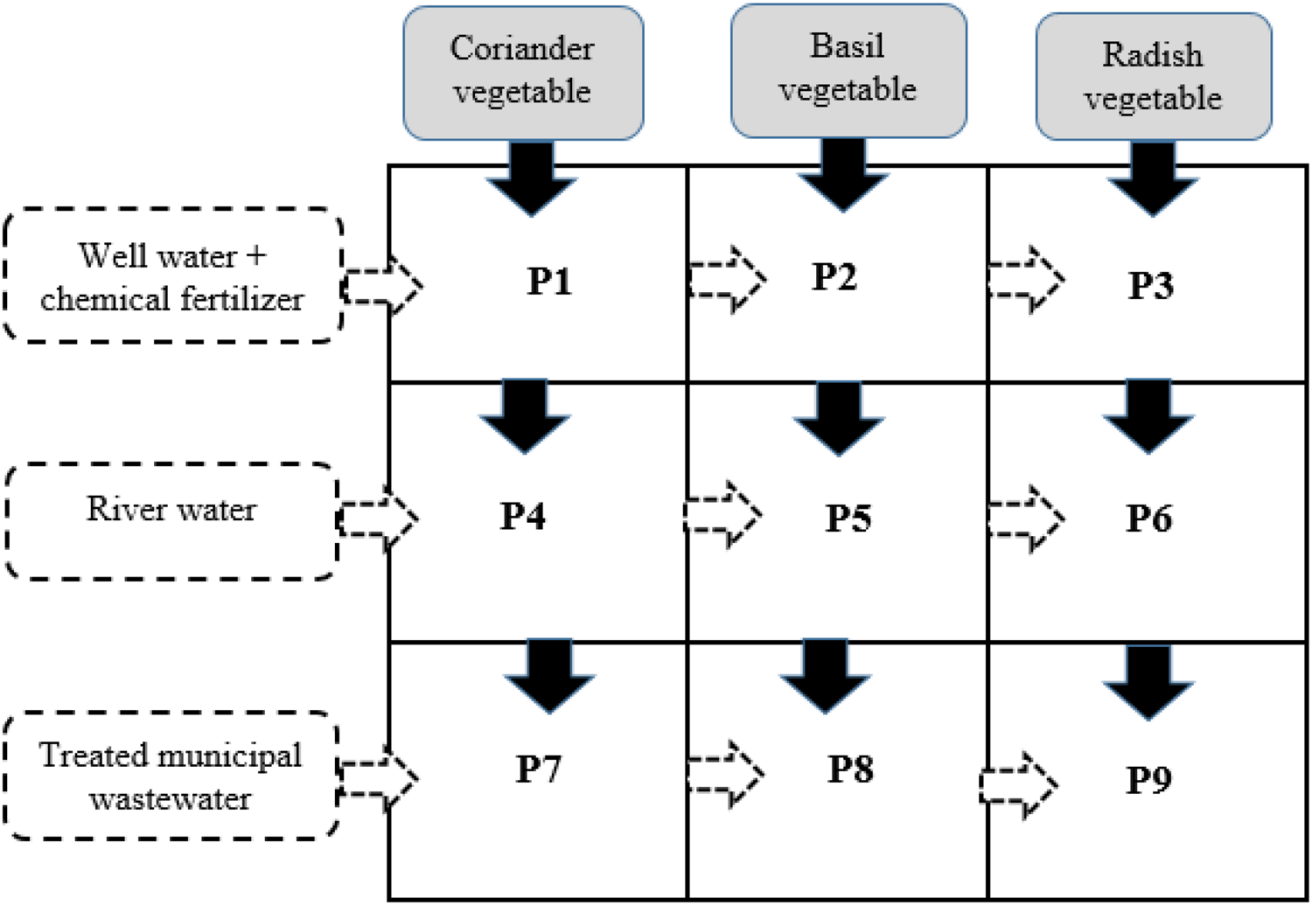
The used patterns for various irrigation methods in the cultivation field.

The cultivation land was divided into three portions (P). The soil used in the farming land was collected from the agricultural farm in Kermanshah city. As depicted in Fig. 2, the three divided parts of the cultivated land were irrigated by three different irrigation water resources as follows:

- First irrigation resource: Well Water (underground water) + nitrogen fertilizer [P1, P2, P3];
- Second irrigation resource: Gharasou River Water [P4, P5, P6];
- Third irrigation resource: Treated Wastewater Effluent of the Kermanshah wastewater treatment plant [P7, P8, P9].

In each of the divided farming land, three types of edible vegetables (Coriander, Basil, and Radish) were planted and irrigated by the mentioned water irrigation type as below:

- Coriander: [P1, P4, and P7]
- Basil: [P2, P5, and P8]
- Radish: [P3, P6, and P9]

After seeding and plowing, all parts were planted for irrigation (Fig. 2). First, irrigated by WW and then for 2 months (60 days) and once every three days, 20 times of irrigation by each source of mentioned irrigations method were applied. Drinking water was used at the cultivation site for WWF irrigation, while 20-l gallons were used to transfer the water needed for irrigation from river water and treated wastewater effluent (TWE)^24^. Furthermore, all parts were irrigated after sunset, lowering the temperature for optimal and valuable irrigation use. P1, P2, and P3 parts were sprayed in three stages throughout the cultivation and irrigation stages for WWF irrigation.

### Reagents

As far as the authors know, all used chemicals in the current study were in analytical grade and used as received without further purification. The nitrogen fertilizer was purchased from a local Iranian brand.

### Sampling

Experimental research and field studies on plants, including the collection of plant material, comply with relevant institutional, national, and international guidelines and legislation^25^. Ten composite samples were taken for each type of irrigation method (WW+F, RW, and TWE); thus, 30 irrigation water samples were analyzed to measure the HMs concentrations^26^. 25 ml of each sample was passed through Whatman® filter paper with a pore size of 42 μm^25^.

Before starting the cultivation process, composite samples were collected and examined to analyze the characteristics and measure the background HMs content of the soil. In this regard, ten composite samples were taken from the soil of the chosen farming land before proceeding with the cultivation process. These samples were collected from 5 points, including P1, P2, P7, P9, and P5 (Fig. 2), so two samples were taken from each section. Samples were taken from the top soil layer (depth of 30 cm) of the corners of each selected section. Soil samples were dried in an ambient condition in the laboratory to remove moisture. Dried soil was crushed by a manual mortar and sieved through 2 mm mesh^27^.

Ten samples were taken from each type of irrigated vegetable, and as a result, 90 samples were collected for analysis. First, the samples were rinsed with tap water to remove mud and external pollution. The rinsed samples were then placed at an ambient temperature in the open air for 24 h to dry. In the next step, the samples were placed in an oven at 60 °C for 24 h to remove moisture (USEPA method 3005 A)^26^. Dried vegetable samples were then pulverized by a shredder and were sieved through 2 mm mesh to remove impurities^28,29^.

#### Acidic digestion of vegetables and soil samples

To digest soil samples, 2 gr of pre-dried soil was added into a balloon (25 ml), then 15 ml of nitric acid (4 N) was added and mixed well. The balloon lid was closed and placed into a hot water bath at 80 °C for 12 h. After 12 h, the balloons were removed from the Ben Marie bath (Labtech, WNB-311 model) to cool to laboratory temperature. After cooling the samples, the samples were filtered with Whatman filter paper with a pore size of 42. Finally, the solution was diluted twice using distilled water (SHWD model)^13,14^.

0.2 g of dried vegetables were weighed and added to a 25 ml balloon to digest vegetable samples. Then 4 ml of nitric acid was added to it. Finally, the balloon lid was closed and placed in a Ben Marie bath (Labtech, WNB-311 model) at 65 °C for 60 min; then, the temperature was raised to 100 °C for another 90 min. Finally, the sample cooling process was done at the ambient temperature of the laboratory, and then 0.2 ml of oxygenated water (37%) was added to digest the organic matter. To complete the process, the samples were rested for half an hour. The final homogenous sample was then passed through the Whatman filter paper. Finally, distilled water diluted the extract twice^30,31^.

#### Measurement of HMs

The HMs content of the vegetables was determined using ICP-OES (SPECTRO, Germany). Limit of detection (LOD) of this device for metals As, Cd, Pb, Cu, Fe, Zn, Cr, Mn, and Ni was 0.179, 0.049, 0.166, 0.306, 0.160, 0.270, 0.564, 0.325, 0.240 parts per million (ppb), respectively. Also, the recovery percentage for the mentioned metals was 96.8 ± 7.2, 98.5 ± 66.6, 94.6 ± 7.2, 104.5 ± 8.4, 97.6 ± 3.3, 101.4 ± 8.4, 95.4 ± 3.6, 98.5 ± 2.8, and 97.5 ± 6.7, respectively.

### Statistical analysis

All statistical analyses were performed using IMB SPSS Statistics version 16.0. One-way variance (ANOVA) was used to compare the mean of each heavy metal among different vegetables and irrigation water types at a significant level (α = 0.05).

### Human health risk assessment

Since the presence of contaminants in vegetables, especially toxic metals, can cause acute and chronic health effects in humans, this study aimed to calculate the health risk posed by As, Pb, and Cd for vegetable consumers^32^. The following calculations were performed for vegetables obtained from all irrigation treatments.

#### Non-carcinogenic risk assessment

Non-carcinogenic risk assessment was performed by the total hazard quotient (THQ) method provided by EPA^33,34^. The HQ stands for Hazard Quotient and is an essential concept in chemical risk assessment. It is applied by regulatory authorities such as U.S.E.P.A to determine the risk category of chemical contaminants. In this method, the estimated daily intake (EDI) was first calculated according to Eq. (1)^35^. Then, considering the oral reference dose, the amount of THQ was obtained based on Eq. (2)^26^.

1 $${\text{CDI}} = \frac{{\left( {{\text{EF}} \times {\text{ED}} \times {\text{IR}} \times {\text{C}}_{{\text{P}}} } \right)}}{{\left( {{\text{BW}} \times {\text{AT}}} \right)}}$$

2 $${\text{THQ}} = \frac{{{\text{EDI}}}}{{{\text{Rfd}}}}$$

A description of each factor related to the Eqs. (1 and 2) was provided in Table 1.

**Table 1:** Description of the necessary factors for health risk assessment of heavy metals.

Parameter	symbol	Unit	Amount
Heavy metals daily intake	EDI	Mg/kg body weight day	Calculated according to Eq. ^1^
Exposure frequency	EF	Day	365
Exposure duration	ED	Year	54
Vegetable ingestion rate	IR	g per person per day	58
Contaminant concentrations in vegetables	CP	Mg/kg dry weight	Concentrations of metals and nitrates based on ICP and spectrophotometry results
Oral reference dose	(Rfd)	Mg/kg body weight	For Pb, As and CD are 03-E5, 04-E3, and 03-E1, respectively For nitrate, this amount is 3.75
Average body weight of consumers	BW	Kg	77.1 ± 14.6
Meantime to produce carcinogenic and non-carcinogenic effects	AT	Day	54 × 365
Cancer slope factor	CSF	No unit	1.5

According to the reports provided by the Iranian Institute of Standards and Industrial Research, the per capita consumption of leafy vegetables is considered to be 58 g person per day^36^. As mentioned earlier, in the current study, the three most consumed types of vegetables among Iranian people (two types of leafy vegetables, Basil and Coriander) and one type of leaf-tuberous vegetable (Radish) have been investigated. Two scenarios were considered for health risk assessment attributed to these three vegetables.

- Scenario 1: In this scenario, it was assumed that the total vegetable consumption (58 g/day) was allocated to only one type of vegetable (Coriander, Basil, or Radish). In this scenario, the contaminant concentration of each vegetable was considered to assess the health risk attributed to each type of vegetable.
- Scenario 2: In this scenario, it was assumed that the total consumption of vegetables (58 g per day) was the sum of these three vegetables. In this scenario, the overall average concentration of contaminants associated with all vegetables was considered to assess the health risk associated with the studied vegetables.

The EPA has declared Rfd for Pb, As, and Cd to be 03-E5-03, 04-E3, and 03-E1 mg/kg body weight, respectively^37,38^. The average body weight of Iranian adults aged 16–70 was 77.45 kg^34^. The amount of AT for non-carcinogenic compounds was considered equal to real-time^39^. This study aimed to assume the worst exposure case to assess the risk of non-carcinogenicity. Therefore, it was considered that Iranian people consumed high vegetables per capita until their 70 s. Eventually, by considering the chosen age range for risk assessment, which was 16–70 years, AT was calculated (54 years × 365 days/year).

Finally, the Total Target Hazard Quotient (TTHQ) was obtained from the total THQ of each metal based on Eq. (3)^40^. If THQ or TTHQ were higher than 1, the result would be considered an acceptable risk for chronic systemic effects. However, if they were higher than 1, the non-carcinogenic risk would be considered unacceptable^37^.

3 $${\text{TTHQ}} = THQ_{As} + THQ_{Pb} + THQ_{Cd } \user2{ }$$

#### Carcinogenic risk assessment

The Incremental Lifetime Cancer Risk (ILCR) calculation method was used for carcinogenic risk evaluation, according to Eq. (4) ^34,37,39^.

4 $$ILCR = {\text{EDI}} \times {\text{CSF }}$$

EDI estimates daily metal intake (mg kg/day), and CSF is the cancer slope factor.

In carcinogenic risk assessment calculations, the considered amount for AT for carcinogenic compounds was 70 years × 365 days/year. According to the US Environmental Protection Agency guideline, the oral CSF parameter was 1.5 mg kg/day. Since the oral CSF for Cd and Pb has not been reported, the ILCR calculation for these metals was avoided and used^39^. Based on the USEPA guidelines, carcinogenic risk < 10^–4^ is acceptable (tolerable). However, if the mentioned risk was > 10^–4^, it could be considered carcinogenic^22,41^.

### Ethical procedure

All experimental research and field studies on plants, including the collection of plant material, comply with relevant institutional, national, and international guidelines and legislation.

## Results and discussion

### Heavy metals in soil and irrigation water sources

The results and characteristics of the soil samples are depicted in Table 2. This table shows that the background concentrations of HMs were less than the announced standard. Based on the results, in the soil of the study site before irrigation, the concentration of non-toxic metals (such as Fe, Zn, Mn, and Cu) was higher than other toxic metals (such as Pb, Cd, and As). The soil's heavy metal content was lower than Iran's national standard. Descriptive parameters related to the evaluation of essential and non-essential metals in the soil of the cultivated area (before vegetables growing) are provided in Table S1 in the supplementary part.

**Table 2:** Background characteristics of the soil samples.

Parameter	Unit	Value	Agricultural soil contamination standard		
			European Union	Iran	
				pH ˂ 7	pH > 7
pH	**–**	7.2–7.4	–	–	–
Background Fe concentration	mg/L	21.614	–	–	–
Background Zn concentration	mg/L	3.236	500	200	300
Background Mn concentration	mg/L	3.033	–	–	–
Background Cu concentration	mg/L	1.046	200	100	140
Background As concentration	mg/L	0.002	40	18	20
Background Pb concentration	mg/L	0.902	75	50	300
Background Cd concentration	mg/L	0.002	5	1	3
Background Cr concentration	mg/L	0.003	110	110	150
Background Ni concentration	mg/L	0.039	110	50	75

Since the most critical challenge that almost all countries face nowadays is food safety and hygiene, thus as a significant contaminant, the HMs content of the consumed vegetables must be regularly analyzed. The results of HMs content are illustrated in Table 3. According to this table, the concentration order of HMs in RW was Fe > Zn > Mn > Ni > Cr > Cu > Pb > As > Cd. This order in TWE was as Fe > Zn > Mn > Cd > Cu > Ni > Pb > Cr > As, and for WW + F was as Fe > Zn > Cr > Ni ≈ Mn > Cu > As ≈ Pb > Cd. In the supplementary part, descriptive parameters related to the evaluation of essential and non-essential metals in the RW, TWE, and WW are provided in Tables S2, S3, and S4.

**Table 3:** Descriptive parameters of various irrigation water sources.

Pollutant	Number of samples	The amount (mg/l)				Standards			
		Mean	SD	Min	Max	FAO	WHO	EPA	IRNDOE
**RW**									
Fe	10	3.7004	1.419	2.020	5.868	5	5	5	3
Zn	10	2.757	1.110	1.391	4.967	2	2	1	2
Mn	10	1.987	0.777	0.974	3.251	0.2	0.2	0.2	1
Cu	10	1.087	0.573	0.435	2.211	0.1	0.2	0.2	0.2
As	10	0.117	0.022	0.085	0.157	0.1	0.1	0.1	0.1
Pb	10	0.858	0.257	0.476	1.240	2	5	5	1
Cd	10	0.082	0.007	0.070	0.093	0.01	0.01	0.01	0.05
Cr	10	1.150	0.082	0.987	1.37	0.1	0.1	0.1	1
Ni	10	1.317	0.238	1.014	1.778	5	0.2	0.2	2
**TWE**									
Fe	10	1.681	0.376	1.035	2.006	5	5	5	3
Zn	10	0.258	0.066	0.143	0.0337	2	2	1	2
Mn	10	0.086	0.062	0.020	0.204	0.2	0.2	0.2	1
Cu	10	0.052	0.028	0.021	0.092	0.1	0.2	0.2	0.2
As	10	0.022	0.007	0.012	0.034	0.1	0.1	0.1	0.1
Pb	10	0.043	0.004	0.037	0.048	2	5	5	1
Cd	10	0.058	0.020	0.034	0.093	0.01	0.01	0.01	0.05
Cr	10	0.029	0.007	0.017	0.040	0.1	0.1	0.1	1
Ni	10	0.045	0.012	0.027	0.062	5	0.2	0.2	2
**WW**									
Fe	10	0.113	0.037	0.060	0.184	5	5	5	3
Zn	10	0.146	0.025	0.108	0.193	2	2	1	2
Mn	10	0.042	0.010	0.025	0.058	0.2	0.2	0.2	1
Cu	10	0.022	0.006	0.009	0.034	0.1	0.2	0.2	0.2
As	10	0.004	0.001	0.003	0.006	0.1	0.1	0.1	0.1
Pb	10	0.004	0.001	0.003	0.005	2	5	5	1
Cd	10	0.002	0.000	0.001	0.002	0.01	0.01	0.01	0.05
Cr	10	0.045	0.004	0.038	0.049	0.1	0.1	0.1	1
Ni	10	0.042	0.007	0.033	0.059	5	0.2	0.2	2

As can be seen, the trend of HMs concentrations in various irrigation sources is different. We assumed that the concentration of essential metals in WW would be higher than that of toxic metals. However, since various HMs can enter natural water resources via domestic wastewater, agricultural runoff, and industrial wastewater, the concentration and order of metals mentioned in TWE and RW would differ from WW. As can be seen in Table 3, the average concentration of Fe, Zn, Mn, Cu, Pb, As, and Cd in RW was the highest, and in WW was the lowest. The results of the ANOVA test revealed that the overall difference between different treatments for metals was significant (P < 0.05).

For Ni, the mean concentration in different types of irrigation water was similar to the above order. However, the mean concentration of Ni between TWE and WW was insignificant (P = 0.997). Cr means in different types of irrigation water was RW > TWE < WW. The mean concentration difference of this metal between TWE and WW was not significant (P = 0.740).

The results showed that the HMs concentration in RW and then TWE was at their highest levels. The possible explanation for massive RW pollution could be that the Gharasou River in Kermanshah is the receiver of various types of treated and sometimes untreated industrial wastewater, which increases the amount of various heavy metals in it.

Also, the river can accept untreated domestic wastewater discharged along the route and upstream. Another possible explanation could be the possibility of entrancing fertilizer-enriched runoffs to the river. While raw domestic wastewater entering the RW can increase the HMs concentration, the treatment processes applied in the wastewater treatment plant reduce the HMs content in the TWE^22,42^.

**Figure 3:**
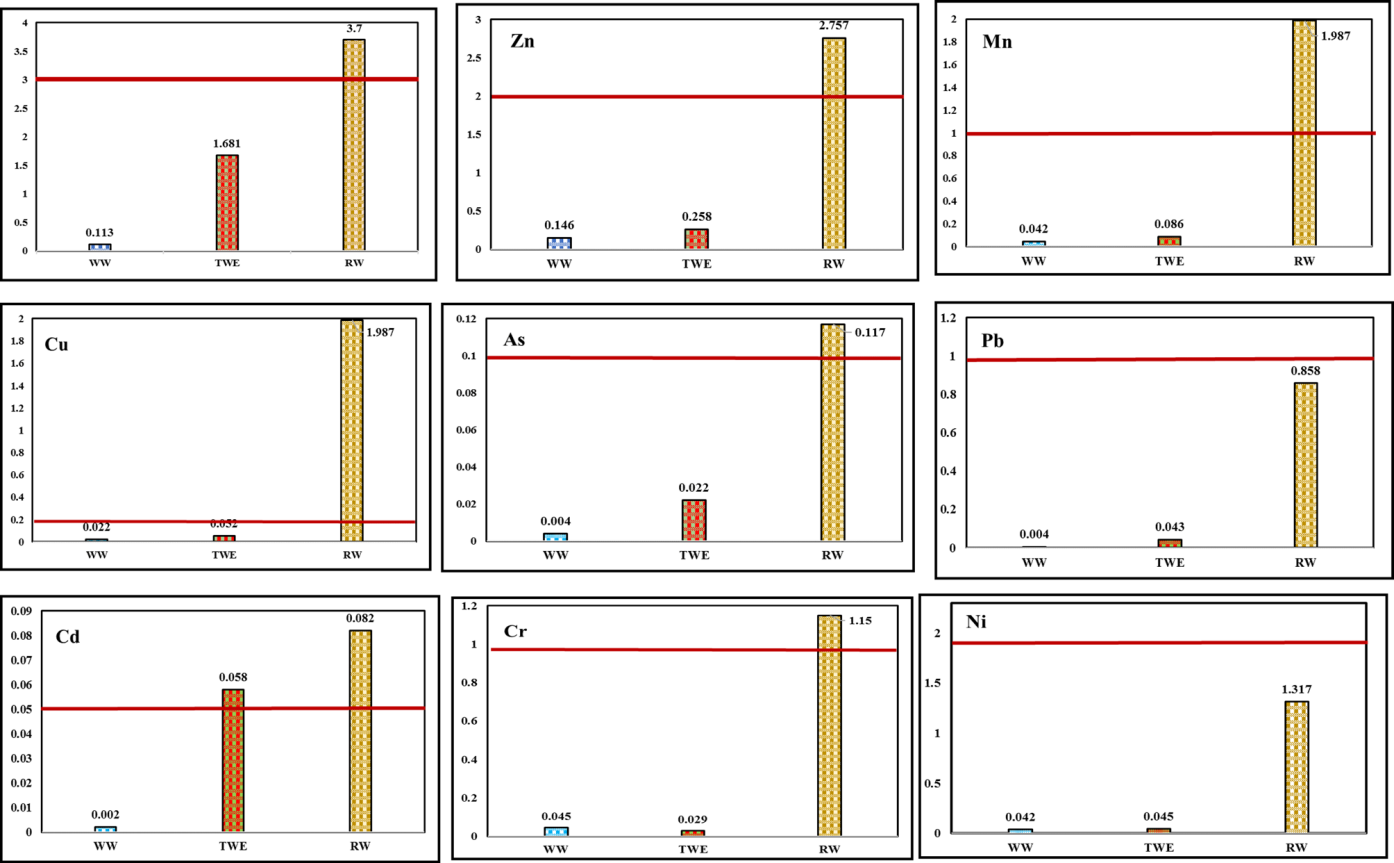
The comparison of the mean average of HMs with IRNDOE standard.

In this study, the quality of different types of water used for irrigation was compared with various irrigation water standards, including FAO^43^, WHO^44^, US-EPA^45^, and the Environmental Protection Agency of Iran (IRNDOE)^46^. The results showed that the highest quality was for WW and the lowest for RW by considering the standards. In WW, the average of all nine metals met all mentioned standards. Although the mean concentration of most measured metals in TWE was higher than WW, the average concentration of all TWE-measured metals (except Cd) was lower than the allowable standard level declared by the EPA^47^. The Cd average in TWE was higher than the allowable level announced by the above organizations. Among the various irrigation water studied, the RW had the lowest quality. The mean concentration of some parameters of this water type (such as Zn, Mn, Cu, As, Cd, and Cr) was higher than the acceptable standard amount announced by all four organizations. While Ni mean concentration was lower than FAO and IRNDOE standards, it was higher than the WHO and EPA standards. In RW, although the Fe and Pb levels in some samples were higher than the standard level, their overall accumulative mean concentration was lower than all the declared standards. It is well-known that plants use photosynthesis process and the main contributor for this process is the Fe metal; this could be the reason why vegetables contain higher contents of Fe^48^. The comparison of the mean average of each HMs with the IRNDOE standard for each irrigation water source is depicted in Fig. 3.

Also, Descriptive parameters related to the concentration of heavy metals in Coriander, Basil, and Radish irrigated with different sources of water irrigation, are provided in Tables S5, S6, and S7 in supplementary part.

### Human health risk assessment

Since there is a possibility that prolonged exposure to toxic metals might have adverse effects on humans, USEPA has introduced carcinogenic and non-carcinogenic parameters to determine health risks related to prolonged exposure to toxic metals^41^.

As depicted in Table 4, the lowest amount of EDI for the As was related to the cultivated Coriander vegetables irrigated with TWE (3.4E^−05^ mg/kg bw day); the highest EDI amount was in the cultivated Radish vegetables irrigated with RW (7.3E^−05^ mg/kg bw day). For Pb, the lowest and highest EDI levels were found in Radish-TWE (6.2 E^−05^ mg/kg bw day) and Basil-RW (2.6 E^−04^ mg/kg bw day), respectively. The lowest EDI for Cd was related to Radish-TWE (4.9 E^−05^ mg/kg bw day), and the highest amount was in Coriander − WW + F (1.5 E^−04^ mg/kg bw day). Factors (e.g., metal concentration, daily consumption of vegetables, and body weight of consumers) can influence obtained EDI amount of each heavy metal. As mentioned before, the considered amount for parameters of vegetable consumption and body weight of consumers were the same in all three types of vegetables, and the only variable parameter in calculating the EDI was the HMs content in vegetables^22,49–51^.

**Table 4:** Daily intake of heavy metals in vegetables irrigated with different sources of irrigation water.

Scenarios	Vegetable type	HMs	Irrigation water sources		
			WW+F	TWE	RW
			EDI (mg/kg bw day)		
First scenario	Coriander	As	4.1 E−05	3.4 E−05	7.0 E−05
		Pb	1.2 E−04	1.1 E−04	8.5 E−05
		Cd	1.5 E−04	8.4 E−05	9.6 E−05
	Basil	As	6.3 E−05	7.2 E−05	1.1 E−04
		Pb	6.5 E−05	1.1 E−04	2.6 E−04
		Cd	7.7 E−05	6.5 E−05	1.3 E−04
	Radish	As	7.3 E−05	6.5 E−05	1.7 E−04
		Pb	1.2 E−04	6.2 E−05	9.3 E−05
		Cd	9.3 E−05	4.9 E−05	1.4 E−04
Second scenario	Total	As	5.9 E−05	5.6 E−05	9.0 E−05
		Pb	1.0 E−04	9.4 E−05	1.7 E−04
		Cd	1.1 E−04	6.6 E−05	1.2 E−04

THQ is related to the non-carcinogenic health risk; the announced acceptable value is ≤ 1^5^. The results of THQ for different irrigation water sources are indicated in Table 5. The results showed that the THQ was different for various HMs based on their irrigation source. The calculated THQ for the vegetables cultivated with WW+F was in the following order: THQ_As_ > THQ_Cd_ > THQ_Pb_. The order of THQ_As_ > THQ_Pb_ > THQ_Cd_ was found in the vegetables cultivated by the TWE resource. According to Table 5, the THQ order in coriander and radish vegetables cultivated by the RW irrigation source was as THQ_As_ > THQ_Cd_ > THQ_Pb_, while for the Basil was THQ_As_ > THQ_Pb_ > THQ_Cd_.

**Table 5:** THQ related to toxic metals through the consumption of irrigated vegetables with different sources of irrigation water.

Scenarios	Vegetable type	HMs	Irrigation water sources		
			WW+F	TWE	RW
			THQ		
First scenario	Coriander	As	0.135	0.113	0.233
		Pb	0.035	0.032	0.024
		Cd	0.149	0.028	0.032
	Basil	As	0.211	0.239	0.345
		Pb	0.018	0.031	0.075
		Cd	0.077	0.022	0.043
	Radish	As	0.243	0.216	0.579
		Pb	0.033	0.018	0.027
		Cd	0.093	0.016	0.046
Second scenario	Total	As	0.196	0.188	0.298
		Pb	0.029	0.027	0.050
		Cd	0.106	0.022	0.040

Due to the similarity of some influential parameters in determining THQ (e.g., consumers' body weight, exposure time, and the number of consumed vegetables), Rfd metals and their measured concentrations in vegetables are the main parameters causing the difference between the obtained THQ^22,52^. Because the Rfd cannot be changed for each metal, and the selected vegetable consumed amount is essential for human health, the only possible way to reduce the THQ is by reducing the vegetables' HMs content. They are several possible ways to mitigate the concentration of toxic metals in crops, such as continuous monitoring and control of contaminants content of the irrigation water sources, prevention of soil contamination, reduction of chemical fertilizers and pesticides, etc.^21,22,49,51,53,54^.

As depicted in Table 5, compared to Cd and Pb, the obtained THQ for the As the metal was significantly higher, which can be due to its higher risk potential^34,41^. The other possible explanation could be its higher concentration than the other two metals in some irrigated vegetables. The results of this study revealed that the calculated THQ for all three HMs in various types of vegetables irrigated by different irrigation sources was lower than the permissible limit (THQ = 1), which was consistent with some similar studies. Cheraghi and Ghobadi et al.^52^ showed that the amount of THQ attributed to Pb metal was higher than the permissible level through the consumption of parsley grown in fields irrigated with TWE . The results of a study conducted by Harmanescu et al.^55^ showed that in vegetables grown in mineral areas, the amount of THQ attributed to Pb metal was higher than the acceptable limit, while the amount of this index for Cd metals was lower than the allowable limit. Research by Wang et al.^56^ in China and another study by Ferré-Huguet N, Martí-Cid R, and Schuhmacher M. 2012 in Spain revealed that the level of THQ for Cd in edible vegetables was lower than the allowable limit.

The possible explanation for the difference between our results and previous studies could be due to the different irrigation water sources, the soil contamination level of vegetables in terms of metals, fertilizer type (chemical or organic), spraying conditions, type of used pesticide, soil characteristics, the characteristic of cultivation site (redox potential, humidity, and pH), geographical conditions, vegetable type, heavy metal concentration in vegetables, weight intended for vegetable consumers, time allotted for exposure to metals through vegetable consumption, etc.^22,52^.

In order to carry out risk management and decide on food safety in terms of non-carcinogenic risk, the total THQ index for all metals (TTHQ) must also be calculated. According to Table 6, the amount of TTHQ related to toxic metals in each vegetable separately, and generally, vegetables cultivated by three irrigation sources were less than the acceptable range (TTHQ = 1).

**Table 6:** TTHQ and ILCR levels of toxic metals through consumption of irrigated vegetables with different sources of irrigation water.

Scenario	Vegetable type	TTHQ/ILCR	Irrigation water sources		
			WW+F	TWE	RW
First scenario	Coriander	TTHQ	0.320	0.173	0.298
		ILCR	6.1 E−05	5.1 E−05	1.0 E−04
	Basil	TTHQ	0.306	0.291	0.472
		ILCR	9.5 E−05	1.1 E−04	1.6 E−04
	Radish	TTHQ	0.369	0.250	0.652
		ILCR	1.1 E−04	9.7 E−05	2.6 E−04
Second scenario	Total	TTHQ	0.331	0.370	0.388
		ILCR	8.8 E−05	8.5 E−05	1.3 E−04

Therefore, consuming vegetables cultivated by all three irrigation treatments does not pose a potential non-carcinogenic risk to consumers. The results found that the ILCR attributed to As in Radish vegetables cultivated by WW+F and Basil vegetables cultivated by the TWE was higher than the allowable Limit (ILCR = 1E^−04^). While in other vegetables cultivated with these two types of irrigation sources, the results were estimated to be less than the allowable limit.

It is a fact that the accumulation of HMs in leafy vegetables is much higher than the fruity vegetables^48,57,58^. Several factors affect the accumulation of HMs in the vegetables, such as environmental location, geological location, and the morphological status of the plants^59^. Considering their morphological status, radish vegetables have tuberous roots, and basil vegetables have long roots, which eventually favors them to have a high potential in absorbing heavy metals (especially for As)^29,51,52,60^. Consequently, if these two types of vegetables grow in soil contaminated with As and irrigation water with a high concentration of As, it can pose a risk to consumers' health^29,51,52^. Ahmadi-Jouibari et al.^22^ showed that the TTHQ level in Coriander, Basil, and Radish vegetables were 0.208, 0.437, and 0.505, respectively. While this value in the present study for vegetables irrigated with WW+F was 0.320, 0.306, and 0.369, respectively, for vegetables irrigated with TWE was 0.173, 0.291, and 0.250, and for RW were 0.289, 0.472, and 0.652, respectively. The main reason for the difference in the results of these two studies was the various concentrations of toxic metals in the studied vegetables, mainly due to the difference in the quality of irrigation water sources and soil of vegetable cultivation. The present study results showed that in all three types of vegetables cultivated by the RW, the ILCR attributed to As was higher than the allowable limit, which is due to higher As content of RW source comparing to the other two resources. The main possible reason for the difference in the results of these two studies was the various concentrations of toxic metals in the studied vegetables, mainly due to the different quality of irrigation water sources and soil of vegetable cultivation. The present study results showed that in all three types of vegetables and the studied vegetables grown with RW, the ILCR attributed to As was higher than the allowable limit.

The possible explanation is that this type of irrigation water source has a higher concentration of As than the other two types of irrigation water sources, so the ILCR rate attributed to As for consumers of all three types of vegetables (even Coriander) was estimated to be higher than the allowable limit. Ahmadi-Jouibari et al.^22^ reported that the ILCR attributed to As due to Coriander, Basil, and Radish vegetables was 8.7E^−05^, 1.9E^−04^, and 2.2E^–04^, respectively. On the other hand, in the present study, these values were 6.1E−^-05^, 9.5E^−05^, and 1.1E^−0.4^ for vegetables irrigated with WW+F, respectively, for vegetables irrigated with TWE was 5.1E^−05^, 1.1E^−0.4^, and 9.7E^−05^, respectively, and for vegetables irrigated with RW was 1.0E^−04^, 1.6E^0.4^, and 2.6E^−0.4^, respectively. The main reason for the difference in the results of these two studies is the diverse concentrations of toxic metals in the studied vegetables, mainly due to the different quality of irrigation water sources and soil of vegetable cultivation. The primary strategy to reduce the risk of carcinogenicity attributed to As in vegetables is to reduce the metal concentration through intervention measures in rice cultivation, such as quality control of irrigation sources, fertilizers, used pesticides, the cultivation of the soil, etc.^22,52^.

## Conclusion

The results of this study showed that, in general, among the source of irrigation water for cultivating vegetables, Gharasou River water has higher pollution in terms of heavy metals than the other two sources (TWE and WW+F). Based on the results, the mean concentrations of each of the metals Pb, Cd, and Pb from highest to lowest was RW > TWE > WW, and the observed difference was statistically significant. Also, the As and Cd in RW and the Cd average in TWE were higher than the irrigation water standards. According to the national standard of Iran, the amount of Cd in all three types of vegetables irrigated with RW and WW+F and the amount of Pb in Basil and Radish vegetables irrigated with RW was higher than the allowable limit. Compared to WHO/FAO standards, only Pb in Basil irrigated with RW was higher than the allowable level. The study results showed that the TTHQ related to toxic metals in vegetables grown with all three irrigation sources was less than acceptable (TTHQ = 1). The present study results showed that the ILCR attributed to As was higher than the allowable limit in all studied vegetables grown with RW. Considering the results obtained in terms of health risks created by heavy metals and compliance of heavy metals in vegetables with national and international standards, it can be concluded that the use of RW Gharasou for irrigation of edible vegetables was not suitable at all. The source of TWE irrigation was preferable to WW+F. In general, it can be said that considering the factors of creating fewer health risks, economic efficiency, no need for chemical and organic fertilizers, abundance, and availability, the best source of irrigation water for growing vegetables in Kermanshah, treated municipal wastewater is the best option.

## Supplementary Information

Supplementary Tables.

## Data Availability

The authors confirm that the data supporting the findings of this study are available within the article and its supplementary materials.
