# Supplementary material for: Investigation of health risk assessment and the effect of various irrigation water on the accumulation of toxic metals in the most widely consumed vegetables in Iran
Source: Sci Rep. 2022 Dec 2;12:20806. doi: 10.1038/s41598-022-25101-9 (PMC9718763; doi:10.1038/s41598-022-25101-9)
Supplement: Supplementary file 1 — Supplementary Tables. [file 41598_2022_25101_MOESM1_ESM.docx]

Supplementary material

| Samples | Fe | Zn | Mn | Cu | As | Pb | Cd | Cr | Ni |
| --- | --- | --- | --- | --- | --- | --- | --- | --- | --- |
| S-1 | 22.656 | 4.291 | 2.946 | 1.344 | 0.003 | 0.926 | 0.002 | 0.003 | 0.042 |
| S-2 | 25.268 | 5.039 | 2.794 | 0.790 | 0.002 | 0.912 | 0.001 | 0.002 | 0.040 |
| S-3 | 23.592 | 2.400 | 4.733 | 1.284 | 0.002 | 0.889 | 0.001 | 0.002 | 0.032 |
| S-4 | 21.379 | 2.192 | 3.345 | 1.133 | 0.001 | 0.908 | 0.002 | 0.006 | 0.035 |
| S-5 | 24.938 | 2.850 | 2.404 | 0.992 | 0.005 | 0.943 | 0.001 | 0.004 | 0.035 |
| S-6 | 18.342 | 2.955 | 3.646 | 0.838 | 0.001 | 0.893 | 0.004 | 0.003 | 0.040 |
| S-7 | 23.945 | 3.894 | 1.874 | 1.043 | 0.001 | 0.901 | 0.007 | 0.002 | 0.038 |
| S-8 | 21.884 | 2.250 | 2.912 | 0.976 | 0.002 | 0.923 | 0.002 | 0.004 | 0.035 |
| S-9 | 19.064 | 3.665 | 2.389 | 1.035 | 0.001 | 0.874 | 0.002 | 0.004 | 0.049 |
| S-10 | 15.068 | 2.826 | 3.280 | 1.025 | 0.001 | 0.853 | 0.002 | 0.002 | 0.046 |
| Mean | 21.614 | 3.236 | 3.033 | 1.046 | 0.002 | 0.902 | 0.002 | 0.003 | 0.039 |
| SD | 3.249 | 0.951 | 0.793 | 0.173 | 0.001 | 0.026 | 0.002 | 0.001 | 0.005 |
| Min | 15.068 | 2.192 | 1.874 | 0.790 | 0.001 | 0.853 | 0.001 | 0.002 | 0.032 |
| Max | 25.268 | 5.039 | 4.733 | 1.284 | 0.005 | 0.943 | 0.007 | 0.006 | 0.049 |

Table S1. Descriptive parameters related to the evaluation of essential and non-essential metals in the soil of the cultivated area (before vegetables growing)

Table S2. Descriptive parameters of River Water (RW)

| Samples | Fe | Zn | Mn | Cu | As | Pb | Cd | Cr | Ni |
| --- | --- | --- | --- | --- | --- | --- | --- | --- | --- |
| S-1 | 4.210 | 2.365 | 1.881 | 1.414 | 0.114 | 0.899 | 0.078 | 1.267 | 1.367 |
| S-2 | 2.103 | 4.003 | 2.460 | 1.250 | 0.102 | 0.476 | 0.093 | 1.117 | 1.447 |
| S-3 | 2.020 | 4.967 | 1.528 | 0.507 | 0.121 | 0.573 | 0.070 | 1.173 | 1.778 |
| S-4 | 2.467 | 1.391 | 2.259 | 1.692 | 0.104 | 0.615 | 0.085 | 1.237 | 1.426 |
| S-5 | 3.860 | 3.637 | 0.974 | 0.543 | 0.128 | 1.100 | 0.089 | 1.137 | 1.087 |
| S-6 | 4.708 | 2.577 | 1.275 | 2.211 | 0.109 | 1.240 | 0.083 | 1.144 | 1.501 |
| S-7 | 3.176 | 2.412 | 1.000 | 0.827 | 0.157 | 1.057 | 0.090 | 0.985 | 1.014 |
| S-8 | 5.765 | 2.045 | 3.251 | 0.844 | 0.100 | 1.054 | 0.081 | 1.073 | 1.301 |
| S-9 | 2.867 | 2.469 | 2.577 | 0.435 | 0.085 | 0.698 | 0.082 | 1.155 | 1.202 |
| S-10 | 5.868 | 1.702 | 2.663 | 1.147 | 0.148 | 0.867 | 0.073 | 1.215 | 1.049 |
| Mean | 3.704 | 2.757 | 1.987 | 1.087 | 0.117 | 0.858 | 0.082 | 1.150 | 1.317 |
| SD | 1.419 | 1.110 | 0.777 | 0.573 | 0.022 | 0.257 | 0.007 | 0.082 | 0.238 |
| Min | 2.020 | 1.391 | 0.974 | 0.435 | 0.085 | 0.476 | 0.070 | 0.985 | 1.014 |
| Max | 5.868 | 4.967 | 3.251 | 2.211 | 0.157 | 1.240 | 0.093 | 1.237 | 1.778 |

Table S3. Descriptive parameters of Treated Wastewater Effluent (TWE)

| Samples | Fe | Zn | Mn | Cu | As | Pb | Cd | Cr | Ni |
| --- | --- | --- | --- | --- | --- | --- | --- | --- | --- |
| S-1 | 2.157 | 0.286 | 0.126 | 0.092 | 0.018 | 0.047 | 0.044 | 0.025 | 0.049 |
| S-2 | 1.525 | 0.278 | 0.050 | 0.043 | 0.024 | 0.037 | 0.076 | 0.040 | 0.062 |
| S-3 | 1.830 | 0.187 | 0.147 | 0.038 | 0.022 | 0.042 | 0.051 | 0.035 | 0.062 |
| S-4 | 1.035 | 0.257 | 0.036 | 0.043 | 0.030 | 0.048 | 0.063 | 0.029 | 0.030 |
| S-5 | 1.046 | 0.337 | 0.020 | 0.021 | 0.015 | 0.045 | 0.084 | 0.028 | 0.027 |
| S-6 | 1.728 | 0.283 | 0.061 | 0.092 | 0.023 | 0.037 | 0.093 | 0.028 | 0.051 |
| S-7 | 2.006 | 0.184 | 0.058 | 0.050 | 0.012 | 0.045 | 0.045 | 0.030 | 0.038 |
| S-8 | 1.762 | 0.143 | 0.204 | 0.021 | 0.018 | 0.044 | 0.045 | 0.020 | 0.053 |
| S-9 | 1.860 | 0.295 | 0.135 | 0.032 | 0.034 | 0.040 | 0.034 | 0.017 | 0.041 |
| S-10 | 1.859 | 0.335 | 0.027 | 0.085 | 0.021 | 0.043 | 0.050 | 0.031 | 0.036 |
| Mean | 1.681 | 0.258 | 0.086 | 0.052 | 0.022 | 0.043 | 0.058 | 0.029 | 0.045 |
| SD | 0.376 | 0.066 | 0.062 | 0.028 | 0.007 | 0.004 | 0.020 | 0.007 | 0.012 |
| Min | 1.035 | 0.143 | 0.020 | 0.021 | 0.012 | 0.037 | 0.034 | 0.017 | 0.027 |
| Max | 2.006 | 0.337 | 0.204 | 0.092 | 0.034 | 0.048 | 0.093 | 0.040 | 0.062 |

Table S4. Descriptive parameters of Well Water (WW)

| Samples | Fe | Zn | Mn | Cu | As | Pb | Cd | Cr | Ni |
| --- | --- | --- | --- | --- | --- | --- | --- | --- | --- |
| S-1 | 0.108 | 0.140 | 0.047 | 0.023 | 0.004 | 0.005 | 0.001 | 0.040 | 0.034 |
| S-2 | 0.060 | 0.151 | 0.025 | 0.021 | 0.005 | 0.004 | 0.002 | 0.047 | 0.043 |
| S-3 | 0.184 | 0.108 | 0.027 | 0.019 | 0.006 | 0.003 | 0.001 | 0.049 | 0.041 |
| S-4 | 0.092 | 0.171 | 0.052 | 0.019 | 0.003 | 0.003 | 0.002 | 0.048 | 0.059 |
| S-5 | 0.154 | 0.193 | 0.058 | 0.023 | 0.003 | 0.005 | 0.002 | 0.049 | 0.045 |
| S-6 | 0.126 | 0.136 | 0.037 | 0.024 | 0.004 | 0.005 | 0.002 | 0.044 | 0.033 |
| S-7 | 0.074 | 0.155 | 0.040 | 0.024 | 0.004 | 0.004 | 0.001 | 0.045 | 0.039 |
| S-8 | 0.129 | 0.120 | 0.043 | 0.026 | 0.004 | 0.004 | 0.002 | 0.045 | 0.043 |
| S-9 | 0.106 | 0.132 | 0.045 | 0.009 | 0.005 | 0.004 | 0.001 | 0.047 | 0.040 |
| S-10 | 0.092 | 0.152 | 0.044 | 0.034 | 0.003 | 0.004 | 0.001 | 0.038 | 0.043 |
| Mean | 0.113 | 0.146 | 0.042 | 0.022 | 0.004 | 0.004 | 0.002 | 0.045 | 0.042 |
| SD | 0.037 | 0.025 | 0.010 | 0.006 | 0.001 | 0.001 | 0.000 | 0.004 | 0.007 |
| Min | 0.060 | 0.108 | 0.025 | 0.009 | 0.003 | 0.003 | 0.001 | 0.038 | 0.033 |
| Max | 0.184 | 0.193 | 0.058 | 0.034 | 0.006 | 0.005 | 0.002 | 0.049 | 0.059 |

Table S5. Descriptive parameters related to the concentration of heavy metals in Coriander, Basil, and Radish irrigated with Waste water (WW) source

| The Coriander irrigated by well water (WW) | | | | | | | | | |
| --- | --- | --- | --- | --- | --- | --- | --- | --- | --- |
| Samples | Fe | Zn | Mn | Cu | As | Pb | Cd | Cr | Ni |
| S-1 | 10.712 | 4.668 | 7.386 | 0.737 | 0.046 | 0.176 | 0.276 | 0.109 | 0.399 |
| S-2 | 10.776 | 4.730 | 6.455 | 0.761 | 0.054 | 0.154 | 0.204 | 0.134 | 0.471 |
| S-3 | 12.687 | 3.295 | 8.346 | 0.592 | 0.050 | 0.168 | 0.264 | 0.154 | 0.397 |
| S-4 | 10.475 | 5.523 | 6.709 | 0.610 | 0.061 | 0.160 | 0.183 | 0.180 | 0.499 |
| S-5 | 10.065 | 4.531 | 6.746 | 0.590 | 0.066 | 0.143 | 0.175 | 0.124 | 0.481 |
| S-6 | 10.113 | 4.271 | 7.449 | 0.578 | 0.041 | 0.181 | 0.172 | 0.118 | 0.430 |
| S-7 | 11.442 | 4.003 | 6.652 | 0.590 | 0.075 | 0.165 | 0.192 | 0.141 | 0.473 |
| S-8 | 9.928 | 3.223 | 6.210 | 0.786 | 0.050 | 0.156 | 0.167 | 0.164 | 0.471 |
| S-9 | 9.274 | 3.662 | 6.104 | 0.667 | 0.042 | 0.162 | 0.152 | 0.142 | 0.408 |
| S-10 | 10.598 | 4.567 | 7.007 | 0.733 | 0.051 | 0.178 | 0.197 | 0.160 | 0.413 |
| Mean | 10.607 | 4.247 | 6.906 | 0.665 | 0.054 | 0.164 | 0.198 | 0.142 | 0.444 |
| SD | 0.934 | 0.713 | 0.672 | 0.082 | 0.011 | 0.012 | 0.041 | 0.022 | 0.039 |
| Min | 9.274 | 3.223 | 6.104 | 0.578 | 0.041 | 0.143 | 0.152 | 0.109 | 0.397 |
| Max | 12.687 | 5.523 | 8.346 | 0.786 | 0.075 | 0.181 | 0.264 | 0.180 | 0.499 |
| The Basil irrigated by well water (WW) | | | | | | | | | |
| Samples | Fe | Zn | Mn | Cu | As | Pb | Cd | Cr | Ni |
| S-1 | 14.347 | 5.511 | 4.051 | 0.506 | 0.101 | 0.085 | 0.105 | 0.121 | 0.347 |
| S-2 | 14.170 | 5.436 | 3.408 | 0.469 | 0.072 | 0.087 | 0.111 | 0.117 | 0.412 |
| S-3 | 14.609 | 5.635 | 3.363 | 0.559 | 0.091 | 0.087 | 0.115 | 0.112 | 0.356 |
| S-4 | 14.061 | 5.250 | 3.570 | 0.500 | 0.069 | 0.097 | 0.102 | 0.116 | 0.363 |
| S-5 | 14.383 | 6.054 | 3.450 | 0.559 | 0.081 | 0.071 | 0.089 | 0.131 | 0.367 |
| S-6 | 15.256 | 5.061 | 3.329 | 0.525 | 0.104 | 0.089 | 0.099 | 0.120 | 0.287 |
| S-7 | 14.992 | 6.117 | 3.419 | 0.472 | 0.072 | 0.076 | 0.091 | 0.103 | 0.324 |
| S-8 | 14.253 | 5.845 | 3.451 | 0.585 | 0.095 | 0.082 | 0.099 | 0.132 | 0.313 |
| S-9 | 14.534 | 5.721 | 3.786 | 0.490 | 0.070 | 0.106 | 0.107 | 0.135 | 0.341 |
| S-10 | 15.064 | 6.019 | 3.752 | 0.594 | 0.085 | 0.080 | 0.100 | 0.132 | 0.350 |
| Mean | 14.567 | 5.665 | 3.558 | 0.526 | 0.084 | 0.086 | 0.102 | 0.122 | 0.346 |
| SD | 0.408 | 0.355 | 0.233 | 0.046 | 0.013 | 0.010 | 0.008 | 0.010 | 0.034 |
| Min | 14.061 | 5.061 | 3.329 | 0.469 | 0.069 | 0.071 | 0.089 | 0.103 | 0.287 |
| Max | 15.256 | 6.117 | 3.786 | 0.594 | 0.104 | 0.106 | 0.115 | 0.135 | 0.412 |
| The Radish irrigated by well water (WW) | | | | | | | | | |
| Samples | Fe | Zn | Mn | Cu | As | Pb | Cd | Cr | Ni |
| S-1 | 20.377 | 7.132 | 6.177 | 0.250 | 0.097 | 0.149 | 0.123 | 0.065 | 0.261 |
| S-2 | 20.341 | 7.095 | 6.177 | 0.348 | 0.092 | 0.157 | 0.123 | 0.075 | 0.249 |
| S-3 | 20.216 | 6.995 | 6.229 | 0.309 | 0.088 | 0.162 | 0.145 | 0.064 | 0.291 |
| S-4 | 19.937 | 7.082 | 6.345 | 0.359 | 0.093 | 0.158 | 0.117 | 0.071 | 0.235 |
| S-5 | 20.108 | 7.203 | 6.046 | 0.293 | 0.098 | 0.171 | 0.109 | 0.071 | 0.232 |
| S-6 | 19.828 | 7.070 | 6.239 | 0.327 | 0.095 | 0.144 | 0.122 | 0.085 | 0.222 |
| S-7 | 20.382 | 7.278 | 6.438 | 0.338 | 0.098 | 0.160 | 0.103 | 0.076 | 0.237 |
| S-8 | 19.568 | 7.209 | 6.175 | 0.350 | 0.098 | 0.144 | 0.138 | 0.063 | 0.215 |
| S-9 | 20.759 | 7.125 | 6.353 | 0.402 | 0.110 | 0.142 | 0.123 | 0.075 | 0.234 |
| S-10 | 19.789 | 7.075 | 6.383 | 0.269 | 0.096 | 0.151 | 0.123 | 0.078 | 0.272 |
| Mean | 20.131 | 7.126 | 6.256 | 0.324 | 0.097 | 0.154 | 0.123 | 0.072 | 0.245 |
| SD | 0.355 | 0.083 | 0.121 | 0.045 | 0.006 | 0.009 | 0.012 | 0.007 | 0.023 |
| Min | 19.568 | 6.995 | 6.046 | 0.250 | 0.088 | 0.142 | 0.103 | 0.063 | 0.215 |
| Max | 20.759 | 7.278 | 6.438 | 0.402 | 0.110 | 0.171 | 0.145 | 0.085 | 0.291 |

Table S6. Descriptive parameters related to the concentration of heavy metals in Coriander, Basil, and Radish irrigated with Treated Wastewater Effluent (TWE) source

| The Coriander irrigated by Treated wastewater effluent (TWE) | | | | | | | | | |
| --- | --- | --- | --- | --- | --- | --- | --- | --- | --- |
| Samples | Fe | Zn | Mn | Cu | As | Pb | Cd | Cr | Ni |
| S-1 | 9.411 | 5.446 | 4.168 | 0.344 | 0.040 | 0.111 | 0.114 | 0.104 | 0.303 |
| S-2 | 8.281 | 5.710 | 4.760 | 0.291 | 0.029 | 0.143 | 0.108 | 0.092 | 0.328 |
| S-3 | 10.715 | 5.125 | 3.925 | 0.366 | 0.050 | 0.194 | 0.110 | 0.102 | 0.342 |
| S-4 | 10.399 | 4.562 | 4.071 | 0.404 | 0.039 | 0.186 | 0.109 | 0.096 | 0.305 |
| S-5 | 11.205 | 5.339 | 4.110 | 0.316 | 0.031 | 0.162 | 0.113 | 0.092 | 0.324 |
| S-6 | 9.099 | 5.077 | 4.413 | 0.319 | 0.068 | 0.140 | 0.116 | 0.098 | 0.291 |
| S-7 | 8.549 | 4.694 | 4.302 | 0.282 | 0.040 | 0.136 | 0.112 | 0.097 | 0.330 |
| S-8 | 9.340 | 4.533 | 3.767 | 0.391 | 0.056 | 0.127 | 0.111 | 0.093 | 0.398 |
| S-9 | 8.763 | 5.684 | 4.125 | 0.327 | 0.044 | 0.179 | 0.125 | 0.102 | 0.340 |
| S-10 | 7.075 | 5.903 | 4.094 | 0.371 | 0.047 | 0.135 | 0.105 | 0.102 | 0.307 |
| Mean | 9.283 | 5.207 | 4.173 | 0.341 | 0.045 | 0.151 | 0.112 | 0.098 | 0.327 |
| SD | 1.235 | 0.494 | 0.273 | 0.041 | 0.012 | 0.027 | 0.005 | 0.004 | 0.030 |
| Min | 7.075 | 4.533 | 3.767 | 0.282 | 0.029 | 0.111 | 0.105 | 0.092 | 0.291 |
| Max | 11.205 | 5.903 | 4.760 | 0.404 | 0.068 | 0.194 | 0.125 | 0.102 | 0.398 |
| The Basil irrigated by Treated wastewater effluent (TWE) | | | | | | | | | |
| Samples | Fe | Zn | Mn | Cu | As | Pb | Cd | Cr | Ni |
| S-1 | 11.790 | 5.081 | 6.859 | 0.693 | 0.093 | 0.146 | 0.090 | 0.176 | 0.584 |
| S-2 | 12.470 | 8.684 | 5.409 | 0.743 | 0.101 | 0.141 | 0.087 | 0.180 | 0.600 |
| S-3 | 14.082 | 6.028 | 5.190 | 0.824 | 0.100 | 0.160 | 0.083 | 0.181 | 0.532 |
| S-4 | 12.264 | 4.821 | 5.864 | 0.716 | 0.084 | 0.133 | 0.092 | 0.185 | 0.566 |
| S-5 | 13.156 | 6.848 | 4.979 | 0.741 | 0.088 | 0.142 | 0.086 | 0.185 | 0.581 |
| S-6 | 13.022 | 7.227 | 5.067 | 0.777 | 0.108 | 0.134 | 0.084 | 0.162 | 0.528 |
| S-7 | 12.535 | 4.662 | 4.938 | 0.736 | 0.090 | 0.142 | 0.087 | 0.195 | 0.537 |
| S-8 | 13.785 | 4.965 | 5.141 | 0.740 | 0.096 | 0.140 | 0.088 | 0.179 | 0.541 |
| S-9 | 11.239 | 7.649 | 4.875 | 0.747 | 0.099 | 0.130 | 0.087 | 0.187 | 0.568 |
| S-10 | 13.454 | 6.703 | 5.903 | 0.693 | 0.091 | 0.155 | 0.085 | 0.197 | 0.548 |
| Mean | 12.780 | 6.267 | 5.422 | 0.741 | 0.095 | 0.142 | 0.087 | 0.183 | 0.559 |
| SD | 0.890 | 1.376 | 0.622 | 0.039 | 0.007 | 0.009 | 0.003 | 0.010 | 0.025 |
| Min | 11.239 | 4.662 | 4.875 | 0.693 | 0.084 | 0.130 | 0.083 | 0.162 | 0.528 |
| Max | 14.082 | 8.684 | 5.903 | 0.824 | 0.108 | 0.160 | 0.092 | 0.197 | 0.600 |
| The Radish irrigated by Treated wastewater effluent (TWE) | | | | | | | | | |
| Samples | Fe | Zn | Mn | Cu | As | Pb | Cd | Cr | Ni |
| S-1 | 19.628 | 3.347 | 5.860 | 0.687 | 0.085 | 0.087 | 0.064 | 0.098 | 0.420 |
| S-2 | 19.306 | 2.819 | 4.522 | 0.647 | 0.078 | 0.076 | 0.073 | 0.087 | 0.434 |
| S-3 | 20.387 | 3.036 | 5.014 | 0.665 | 0.080 | 0.078 | 0.074 | 0.095 | 0.487 |
| S-4 | 19.739 | 3.064 | 5.790 | 0.657 | 0.087 | 0.088 | 0.065 | 0.092 | 0.468 |
| S-5 | 19.230 | 3.267 | 5.041 | 0.704 | 0.081 | 0.078 | 0.084 | 0.102 | 0.455 |
| S-6 | 19.360 | 3.342 | 4.955 | 0.674 | 0.089 | 0.087 | 0.058 | 0.094 | 0.439 |
| S-7 | 18.096 | 3.102 | 5.610 | 0.677 | 0.094 | 0.099 | 0.066 | 0.097 | 0.478 |
| S-8 | 19.191 | 3.150 | 5.028 | 0.663 | 0.083 | 0.079 | 0.059 | 0.105 | 0.432 |
| S-9 | 18.830 | 3.235 | 4.942 | 0.679 | 0.099 | 0.085 | 0.052 | 0.097 | 0.448 |
| S-10 | 17.725 | 3.385 | 4.467 | 0.674 | 0.084 | 0.069 | 0.056 | 0.105 | 0.418 |
| Mean | 19.149 | 3.175 | 5.123 | 0.673 | 0.086 | 0.083 | 0.065 | 0.097 | 0.448 |
| SD | 0.777 | 0.176 | 0.484 | 0.016 | 0.006 | 0.008 | 0.010 | 0.006 | 0.024 |
| Min | 17.725 | 2.819 | 4.467 | 0.647 | 0.078 | 0.069 | 0.052 | 0.087 | 0.418 |
| Max | 20.387 | 3.385 | 5.790 | 0.704 | 0.099 | 0.099 | 0.084 | 0.105 | 0.487 |

Table S7. Descriptive parameters related to the concentration of heavy metals in Coriander, Basil, and Radish irrigated with River Water (RW) source

| The Coriander irrigated by River water (RW) | | | | | | | | | |
| --- | --- | --- | --- | --- | --- | --- | --- | --- | --- |
| Samples | Fe | Zn | Mn | Cu | As | Pb | Cd | Cr | Ni |
| S-1 | 18.431 | 4.459 | 6.260 | 1.963 | 0.096 | 0.122 | 0.076 | 0.286 | 0.223 |
| S-2 | 19.147 | 4.575 | 6.645 | 1.739 | 0.102 | 0.080 | 0.160 | 0.200 | 0.279 |
| S-3 | 19.350 | 5.767 | 7.055 | 2.676 | 0.067 | 0.109 | 0.099 | 0.241 | 0.267 |
| S-4 | 18.160 | 4.588 | 3.884 | 1.150 | 0.103 | 0.134 | 0.196 | 0.262 | 0.224 |
| S-5 | 18.154 | 4.700 | 6.142 | 2.013 | 0.073 | 0.115 | 0.151 | 0.281 | 0.222 |
| S-6 | 19.546 | 8.138 | 6.953 | 1.636 | 0.105 | 0.113 | 0.093 | 0.236 | 0.236 |
| S-7 | 17.252 | 5.249 | 7.182 | 2.227 | 0.079 | 0.133 | 0.094 | 0.220 | 0.234 |
| S-8 | 18.348 | 4.308 | 5.482 | 1.830 | 0.089 | 0.109 | 0.137 | 0.219 | 0.184 |
| S-9 | 18.954 | 4.605 | 6.080 | 1.855 | 0.101 | 0.105 | 0.155 | 0.225 | 0.256 |
| S-10 | 18.325 | 5.206 | 6.920 | 2.275 | 0.117 | 0.105 | 0.111 | 0.177 | 0.277 |
| Mean | 18.567 | 5.160 | 6.260 | 1.936 | 0.093 | 0.113 | 0.127 | 0.235 | 0.240 |
| SD | 0.687 | 1.138 | 0.991 | 0.411 | 0.016 | 0.015 | 0.038 | 0.034 | 0.030 |
| Min | 17.252 | 4.308 | 3.884 | 1.150 | 0.067 | 0.080 | 0.076 | 0.177 | 0.184 |
| Max | 19.546 | 8.138 | 7.182 | 2.676 | 0.117 | 0.134 | 0.196 | 0.281 | 0.279 |
| The Basil irrigated by River water (RW) | | | | | | | | | |
| Samples | Fe | Zn | Mn | Cu | As | Pb | Cd | Cr | Ni |
| S-1 | 25.598 | 6.629 | 7.017 | 1.056 | 0.131 | 0.329 | 0.183 | 0.585 | 1.104 |
| S-2 | 26.165 | 6.893 | 6.829 | 1.025 | 0.168 | 0.372 | 0.151 | 0.659 | 1.155 |
| S-3 | 25.652 | 6.530 | 7.555 | 0.976 | 0.138 | 0.353 | 0.152 | 0.626 | 1.136 |
| S-4 | 25.845 | 7.023 | 7.513 | 0.892 | 0.121 | 0.345 | 0.224 | 0.668 | 1.137 |
| S-5 | 25.782 | 6.744 | 7.054 | 1.012 | 0.166 | 0.369 | 0.182 | 0.630 | 1.170 |
| S-6 | 25.948 | 6.677 | 7.024 | 0.991 | 0.125 | 0.386 | 0.189 | 0.613 | 1.145 |
| S-7 | 25.458 | 6.748 | 7.301 | 0.964 | 0.139 | 0.292 | 0.191 | 0.690 | 1.121 |
| S-8 | 25.802 | 6.680 | 7.113 | 0.902 | 0.146 | 0.344 | 0.203 | 0.550 | 1.150 |
| S-9 | 26.195 | 6.582 | 7.019 | 0.972 | 0.132 | 0.330 | 0.129 | 0.626 | 1.175 |
| S-10 | 26.199 | 6.778 | 7.106 | 1.174 | 0.145 | 0.382 | 0.130 | 0.687 | 1.116 |
| Mean | 25.864 | 6.728 | 7.153 | 0.996 | 0.141 | 0.350 | 0.173 | 0.633 | 1.141 |
| SD | 0.260 | 0.146 | 0.232 | 0.080 | 0.016 | 0.029 | 0.031 | 0.045 | 0.023 |
| Min | 25.458 | 6.530 | 6.829 | 0.892 | 0.121 | 0.292 | 0.129 | 0.550 | 1.104 |
| Max | 26.199 | 7.023 | 7.555 | 1.174 | 0.168 | 0.386 | 0.224 | 0.690 | 1.175 |
| The Radish irrigated by River water (RW) | | | | | | | | | |
| Samples | Fe | Zn | Mn | Cu | As | Pb | Cd | Cr | Ni |
| S-1 | 26.852 | 6.800 | 4.106 | 1.434 | 0.124 | 0.226 | 0.186 | 0.554 | 0.339 |
| S-2 | 23.242 | 6.924 | 4.206 | 1.432 | 0.127 | 0.232 | 0.179 | 0.569 | 0.327 |
| S-3 | 24.045 | 6.842 | 4.107 | 1.442 | 0.125 | 0.221 | 0.176 | 0.564 | 0.305 |
| S-4 | 24.574 | 6.590 | 4.196 | 1.456 | 0.122 | 0.227 | 0.191 | 0.548 | 0.321 |
| S-5 | 23.061 | 6.750 | 4.061 | 1.450 | 0.118 | 0.223 | 0.179 | 0.571 | 0.309 |
| S-6 | 26.185 | 6.604 | 4.158 | 1.452 | 0.119 | 0.238 | 0.181 | 0.567 | 0.314 |
| S-7 | 26.168 | 6.532 | 4.121 | 1.437 | 0.134 | 0.232 | 0.179 | 0.558 | 0.314 |
| S-8 | 26.429 | 7.141 | 4.124 | 1.441 | 0.117 | 0.227 | 0.183 | 0.566 | 0.320 |
| S-9 | 26.280 | 6.759 | 4.077 | 1.436 | 0.132 | 0.236 | 0.199 | 0.551 | 0.354 |
| S-10 | 26.518 | 6.564 | 4.004 | 1.441 | 0.117 | 0.252 | 0.183 | 0.556 | 0.321 |
| Mean | 25.335 | 6.751 | 4.116 | 1.442 | 0.124 | 0.231 | 0.184 | 0.560 | 0.322 |
| SD | 1.453 | 0.189 | 0.061 | 0.008 | 0.006 | 0.009 | 0.007 | 0.008 | 0.015 |
| Min | 23.061 | 6.532 | 4.004 | 1.432 | 0.117 | 0.221 | 0.176 | 0.548 | 0.305 |
| Max | 26.518 | 7.141 | 4.206 | 1.456 | 0.134 | 0.252 | 0.199 | 0.571 | 0.354 |
